# Comparison of anesthetic effects of xylazine combined with alfaxalone or ketamine and maintained with isoflurane in captive Formosan Reeve’s Muntjac (*Muntiacus reevesi micrurus*)

**DOI:** 10.1186/s13620-025-00291-6

**Published:** 2025-02-08

**Authors:** Li-Jen Chang, Zixuan Wang, Chen-Yeh Lien, Amanda H.C. Wen

**Affiliations:** 1 Department of Small Animal Clinical Science, Virginia-Maryland College of Veterinary Medicine, Blacksburg, VA 24061 USA; 2 College of Veterinary Medicine, China Agricultural University, Beijing, 100193 China; 3 National Key Laboratory of Veterinary Public Health and Safety, China Agricultural University, Beijing, 100193 China; 4 Animal Medical Center, Taipei Zoo, Taipei, 11656 Taiwan; 5 Central Florida Zoo, Sanford, FL 32819 USA

**Keywords:** Alfaxalone, Ketamine, Xylazine, Formosan Reeve's muntjac, Anesthesia

## Abstract

Formosan Reeve’s muntjac is a Cervidae species endemic to Southeast China and Taiwan. However, research on different anesthetic protocols, their effects, and their safety in Formosan Reeve’s muntjac is limited. This study evaluated the effects of ketamine-xylazine (KX) and alfaxalone-xylazine (AX) administered via blow darts to nine muntjacs. Induction and recovery times as well as the quality were assessed by a blinded observer. Peripheral oxygen saturation (SpO_2_), heart rate, respiratory rate, and rectal temperature were recorded for at least 30 min. Tolazoline (4 mg/kg) was used post-procedure to reverse xylazine’s effects. The mean doses were 4.68 ± 2.18 mg/kg for ketamine and 3.22 ± 1.33 mg/kg for xylazine in the KX group. In the AX group, the mean doses were 4.38 ± 0.31 mg/kg for alfaxalone and 1.19 ± 0.26 mg/kg for xylazine. The median induction times were 339.5 s (range 180.0-375.0) for KX and 125.0 s (range 71.0–334.0) for AX, with both groups scoring 3.0 for induction quality. The recovery times were 507.5 s (range 266.0–1081.0) for KX and 243.0 s (range 92.0–480.0) for AX, with recovery scores of 2.3 and 3.0, respectively, showing no significant difference. Hypoxemia (SpO_2_ < 90%) was more severe in the KX group when compared to the AX group (SpO_2_ > 92%), and rectal temperatures were higher in the former during the first 15 min. Heart and respiratory rates showed no significant differences between groups. Our findings demonstrate that both anesthetic combinations achieve reliable induction and satisfactory recovery in Formosan Reeve’s muntjac, with the ketamine-xylazine combination causing a more profound hypoxemia post-induction compared to the alfaxalone-xylazine combination.

## Introduction

Formosan Reeve’s muntjac (*Muntiacus reevesi micrurus*) is a small wild cervid species endemic to the subtropical and tropical forests of southeast China and Taiwan [1]. Adult males typically weigh approximately 10 kg, whereas females weigh approximately 8 kg [2]. Like many prey species, Formosan Reeve’s muntjacs exhibit a significant stress response when restrained or pursued, making them more susceptible to anesthesia-related complications, including ruminal tympany, hypoxemia, and capture myopathy [3]. Therefore, smooth induction and stable maintenance of anesthesia are crucial for the management, care, and welfare of these animals.

However, research on different anesthetic protocols, their effects, and their safety in Formosan Reeve’s muntjac is limited. Intramuscular injections of butorphanol, azaperone, and medetomidine, initially used for white-tailed deer anesthesia, have been applied to Reeve’s muntjac for short-duration anesthesia [4, 5]. Alpha-2 adrenergic agonists, such as xylazine, dexmedetomidine, and medetomidine, are widely used in wildlife anesthesia owing to their efficacy and ease of reversal; however, these are notorious for their significant cardiovascular side effects, such as vasoconstriction and bradycardia [6]. Xylazine, a relatively nonselective alpha-2 agonist, has been used in veterinary medicine for decades [7]. It has been employed in various diagnostic, therapeutic, and research procedures involving cervids, including white-tailed deer, black-tailed deer, and Formosan sika deer, with dosages ranging from 0.10 to 10.0 mg/kg depending on the purpose of sedation and the species [8–10]. Although xylazine produces sedation, analgesia, and muscle relaxation, it also has dose-dependent adverse effects such as respiratory depression, hypothermia, anorexia, and salivation [8, 11]. Consequently, reversal agents are necessary to mitigate the undesirable side effects of xylazine [3]. Yohimbine and tolazoline have been demonstrated to act as effective antagonists of xylazine in many deer species [12, 13].

Ketamine is a widely used dissociative anesthetic, providing rapid anesthesia, analgesia and relative cardiovascular stability through sympathetic stimulation [14]. It can be administered intravenously or intramuscularly. However, ketamine can cause undesirable side effects, including hyperthermia, dysphoria, vocalization, and ataxia during recovery [15]. Combining ketamine with alpha-2 agonists can enhance the quality of induction and recovery by providing better analgesia and muscle relaxation [3, 16]. The ketamine-xylazine (KX) combination has been extensively used for reliable immobilization in many cervid species, including white-tailed, Axis, Javan Rusa, desert mule, and Formosan sika deer [10, 17–20]. Despite the reversibility of alpha-2 agonist effects, issues such as dysphoria and poor recovery persist when using the KX combination due to dissociative-associated side effects [6]. Additionally, xylazine-induced respiratory depression can be exacerbated when combined with ketamine, increasing the severity of hypoxemia during anesthesia [21].

Alfaxalone is a neuroactive-steroid molecule capable to induce anesthesia, which is currently available as a 1% solution in 2-hydroxypropyl-β-cyclodextrin (alfaxalone-HPCD) [22]. It has been used in dogs, cats, amphibians, reptiles, and avian species since its first release in veterinary market [22–25]. Alfaxalone offers advantages such as flexible administration routes, rapid onset, excellent muscle relaxation, quiet recovery, and cardiovascular stability [23, 26]. However, it has minimal analgesic effects and is often combined with other tranquilizers, sedatives, or anesthetics for pre-medication or induction [27]. In dogs and cats, alfaxalone is frequently administered with opioids, benzodiazepines, and/or alpha-2 agonists for various procedures [28, 29]. The medetomidine-azaperone-alfaxalone (MAA) combination has been successfully used to induce anesthesia in mule deer, white-tailed deer, and elk [6, 30, 31]. However, using alfaxalone for wild cervid anesthesia can be challenging due to the larger injection volumes required compared with ketamine-based combinations, especially when remote delivery is needed [32]. Darting is a common method for immobilizing free-ranging animals in zoos and the wild, whereby the injection volume and physical properties of the drug affect the accuracy and reliability of delivery [33]. The commercially available form of alfaxalone is a 10 mg/mL solution (Alfaxan^®^), which may impact drug delivery due to its low concentration [34].

This study aimed to evaluate and compare the effects of intramuscular ketamine-xylazine (KX) or alfaxalone-xylazine (AX) induction, followed by isoflurane maintenance, in captive Reeve’s muntjac. The time and quality of induction and recovery was compared. Additionally, we aimed to assess the effects of KX and AX combinations on heart rate (HR), respiratory rate (RR), hemoglobin oxygen saturation (SpO_2_), and rectal temperature (RT) during general anesthesia.

## Materials and methods

### Animals

Nine adult Formosan Reeve’s muntjacs (five males and four females) housed at Taipei Zoo, Taipei, Taiwan (24°59’42” N, 121°35’3” E) were included in this study. The study protocol was approved by the Institutional Animal Care and Use Committee of the Taipei Zoo (protocol code 10502). The purpose of the anesthesia was to conduct annual health examinations. This was a single-blinded, randomized clinical trial. The physiological status of each animal was evaluated based on American Society of Anesthesiologists Physical Status (ASA-PS) classification system. All animals were assigned ASA-PS I or II, which is associated with relatively lower anesthetic risks [35]. Food and water were withheld for at least 12 h before anesthesia.

### Anesthesia

The anesthesia protocol was determined by lottery. The muntjacs received either a KX or AX combination for anesthesia. Both combinations were administered intramuscularly via a blow dart loaded into a 3-ml (KX) or 5-ml (AX) syringe by two well-trained and experienced veterinarians (LJC and CYL). Four muntjacs were anesthetized with the KX combination (Imaldene 1000^®^; Merial, Taipei, Taiwan, and Balazine^®^ 2%; Health-tech Pharmaceutical, Taipei, Taiwan), while the other five were anesthetized with the AX combination (Alfaxan^®^; E-Rei, Taipei, Taiwan). The anesthetic dosage was calculated based on body weight from visual estimates and previous medical records, also incorporating temperament, such as stress, fear, anxiety, and aggression. The reference dosages of xylazine and ketamine were 2–3 mg/kg and 4–6 mg/kg, respectively [10, 36]. Alfaxalone dosages were determined in a pilot study, which determined the appropriate dose as 3–4 mg/kg. Exact body weights were measured after induction to calculate the drug dose accurately. Induction time was defined as the period between the administration of anesthetics and the attainment of lateral recumbency, which was timed and recorded. Induction quality was assessed and recorded by a blinded observer (AW) using a scoring system detailed in Table 1. The muntjacs were positioned in sternal recumbency and immediately transported to the animal medical center of Taipei Zoo, a process which took 5–10 min. The anesthetized animals were kept in sternal recumbency with the neck elevated and nostrils pointing downward to reduce the risk of aspiration pneumonia. 100% of oxygen from an E tank was delivered at a rate of 10 L/min via nasal insufflation during transportation. The muntjacs’ tracheas were intubated with 4–5 mm (I.D.) cuffed endotracheal tubes (Medline, Fort Pierce, USA), depending on their body weight. If the first intubation attempt failed, isoflurane (5%) with oxygen was administered via a mask to facilitate intubation. An intravenous catheter was placed in the cephalic vein using a 22-gauge IV catheter (Terumo, Taipei, Taiwan). Isoflurane was used for anesthetic maintenance via a rebreathing circuit with an oxygen flow rate of 20–40 mL/kg/min. The vaporizer was set between 1.5 and 2.5% based on the depth of anesthesia of the individual. The depth of anesthesia was assessed by the veterinarian based on the palpebral reflex, eyeball position, and jaw tone. The muntjacs breathed spontaneously throughout the procedure. Isoflurane and oxygen were delivered using a Datex-Ohmeda Excel 210SE^®^ anesthesia machine (Ohmeda, Madison, WI, USA).

**Table 1:** Anesthetic quality Scoring System (AQSS) definition chart

Score	Induction	Recovery
0	High excitement (vocalizes, jumps, or attempts to escape during physical restraint, unable to place the endotracheal tube)	Rough (several uncoordinated attempts to stand, ataxic)
1	Moderate excitement (some struggling, falling, or slipping after becoming recumbent; able to place the endotracheal tube)	Relatively rough (several coordinated attempts to stand, ataxic)
2	Low excitement (some struggling, no falling or slipping; may or may not be intubated within 60 s)	Relatively calm (1–2 coordinated attempts to stand with minimal short-lived ataxia)
3	Excitement-free induction (no outward signs of excitement, tracheal intubation easy)	Excitement-free recovery (no outward signs of excitement, stood up smoothly and quickly)

### Assessment of physiological indices and recovery

**Fig. 1:**
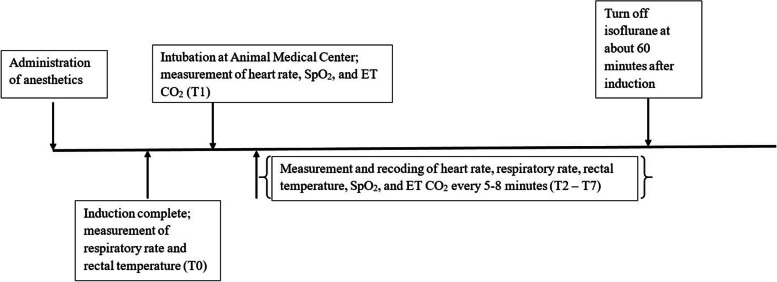
Timeline illustration of the study. The time points and associated events are shown. A total of 9 Reeve’s muntjacs were recruited in this study. Four animals were assigned to ketamine-xylazine (KX) group (mean dose of ketamine at 4.68 ± 2.18 mg/kg, mean dose of xylazine at 3.22 ± 1.33 mg/kg) and the other five were assigned to alfaxalone-xylazine (AX) group (mean dose of alfaxalone at 4.38 ± 0.31 mg/kg, mean dose of xylazine at 1.19 ± 0.26 mg/kg). Both combinations were administered intramuscularly via a blow dart

Heart rate (HR), respiratory rate (RR), oxygen saturation (SpO_2_), and rectal temperature (RT) were monitored and recorded every 5–8 min during the procedure. Respiratory rate and rectal temperature were recorded after induction (T0). T1 was marked as the time point immediately after intubation when other physiological parameters started to be recorded, with subsequent time points at 5–8 min intervals during the procedure. Peripheral pulse oxygen saturation was measured using the Masimo Rad-5^®^ pulse oximeter (Masimo, Irvine, CA, USA), and the probe was placed on the tongue. Hypoxemia was defined as SpO_2_ less than 90% [37]. The muntjacs were warmed with a circulating water heating pad (Gaymar Stryker^®^ TP 650, MI, USA) if the first measured rectal temperature was below 37.5℃. Upon completion of the procedure, isoflurane was discontinued, and the recording was ceased. The timeline of the study is shown in Fig. 1. The muntjacs were relocated to their enclosure for recovery. An endotracheal tube remained in place during transportation, and the animals were kept in sternal recumbency with 20–40 mL/kg/min of oxygen supplied via the endotracheal tube. Tolazoline at 4 mg/kg was administered intravenously to the muntjacs to reverse the effects of xylazine after the procedure. Recovery time was defined as the period between tolazoline injection and the animal standing on all four feet. Recovery quality was assessed and recorded by a blinded observer (AW) using criteria listed in Table 1. The recovery scoring system was adapted from a previous study [38], whereas the induction scoring criteria were established by the authors of this study.

### Statistical analysis

Normal probability plots were inspected to assess the data distribution. The dosage of each anesthetic, including xylazine, ketamine, alfaxalone, and tolazoline, was calculated based on the exact body weight of the animals. Results are presented as mean ± SD or median and range, as appropriate. Boxplots with individual data points were used to visualize the distribution of induction time, induction score, recovery time, and recovery score. The Wilcoxon rank-sum test was employed to detect differences between groups. Additionally, a mixed model analysis of variance (ANOVA) was conducted to determine differences in physiological parameters between the groups. SAS software (Version 9.4, Cary, NC, USA) was used for analyzing the data. Statistical significance was set at *p* < 0.05.

## Results

The duration of anesthesia was approximately 60 min. No instances of regurgitation, aspiration pneumonia, bloating, or other major anesthesia-associated complications were observed throughout the procedure. Apneustic breathing pattern was observed in all animals (4/4) in the KX group after induction. The tracheas of all Reeve’s muntjacs were successfully intubated on the first attempt, and all subjects recovered without any issues.

The mean body weight of Reeve’s muntjacs was 8.63 ± 1.77 kg, ranging from 4.04 kg to 10.61 kg. In the KX group, the mean doses were 4.68 ± 2.18 mg/kg for ketamine and 3.22 ± 1.33 mg/kg for xylazine. In the AX group, the mean doses were 4.38 ± 0.31 mg/kg for alfaxalone and 1.19 ± 0.26 mg/kg for xylazine.

**Fig. 2:**
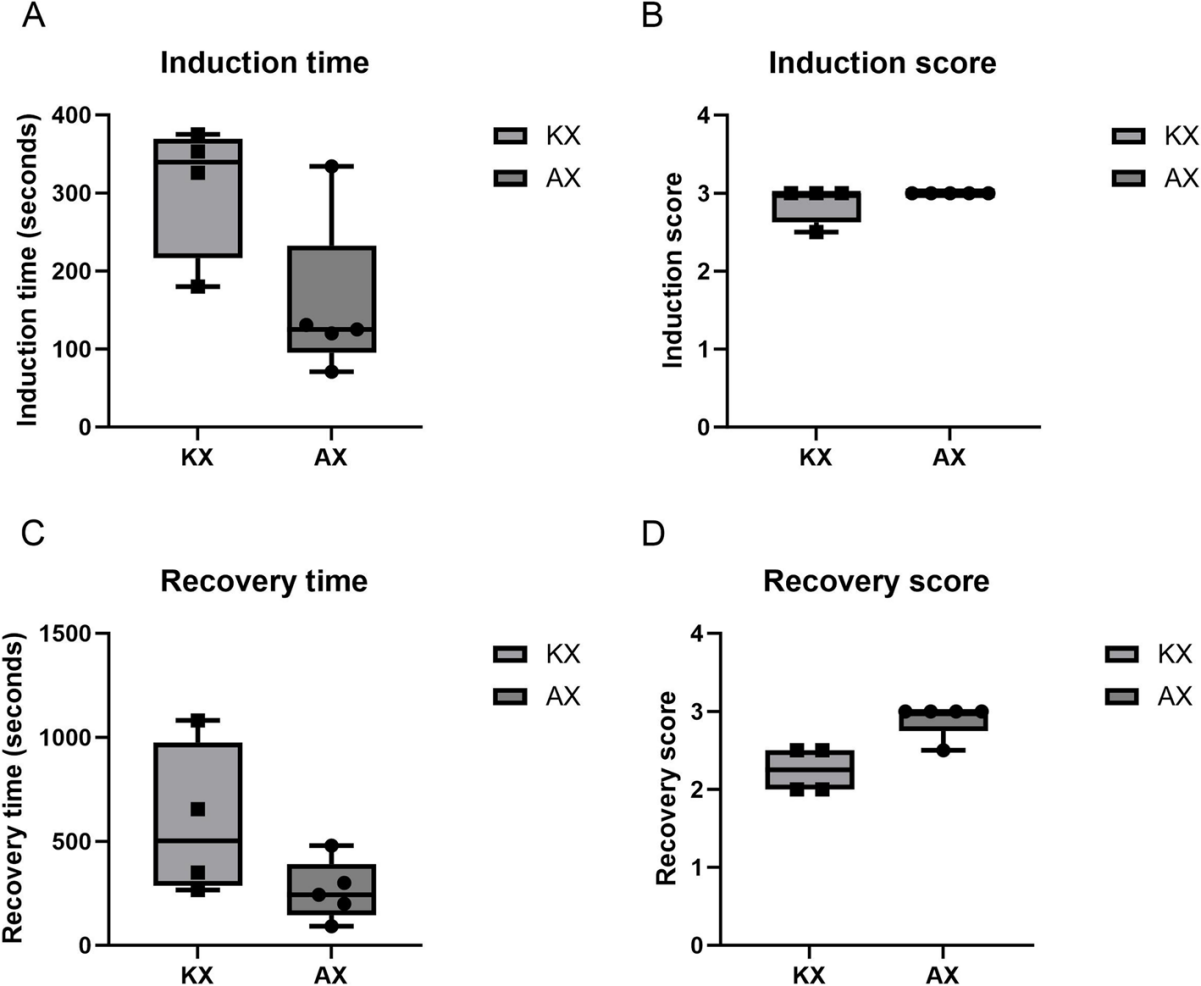
Boxplots depicting the induction time (**A**), induction score (**B**), recovery time (**C**), and recovery score (**D**) for the ketamine-xylazine (KX) group (*n* = 4, mean dose of ketamine at 4.68 ± 2.18 mg/kg, mean dose of xylazine at 3.22 ± 1.33 mg/kg) and alfaxalone-xylazine (AX) group (*n* = 5, mean dose of alfaxalone at 4.38 ± 0.31 mg/kg, mean dose of xylazine at 1.19 ± 0.26 mg/kg). Both combinations were administered intramuscularly via a blow dart. Each dot represents an individual measurement, while the inner horizontal lines indicate the median values

The median induction time was 339.5 s (range 180.0–375.0) for the KX group and 125.0 s (range 71.0–334.0) for the AX group (*p* = 0.1035) (Fig. 2A). The median induction score was 3.0 for both groups, with a range of 2.5–3.0 for the KX group and 3.0–3.0 for the AX group (*p* = 0.3972) (Fig. 2B). The median recovery time was 507.5 s (range 266.0–1081.0) for the KX group and 243.0 s (range 92.0–480.0) for the AX group (*p* = 0.1500) (Fig. 2C). The median recovery score was 2.3 (range 2.0–2.5) for the KX group and 3.0 (range 2.5–3.0) for the AX group (*p* = 0.0567) (Fig. 2D). No significant differences in induction time, induction score, recovery time, or recovery score were detected between the groups.

**Fig. 3:**
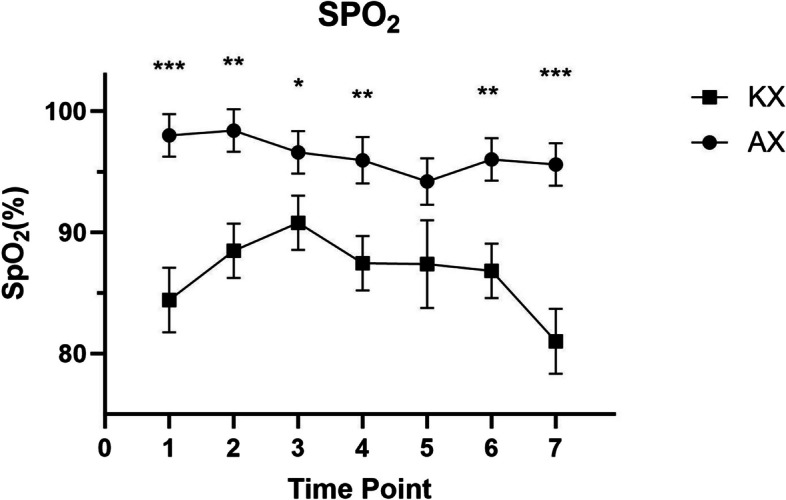
Peripheral oxygen saturation (SpO2) levels in Reeve’s Muntjac administered intramuscular ketamine-xylazine (KX) (*n* = 4, mean dose of ketamine at 4.68 ± 2.18 mg/kg, mean dose of xylazine at 3.22 ± 1.33 mg/kg) and alfaxalone-xylazine (AX) (*n* = 5, mean dose of alfaxalone at 4.38 ± 0.31 mg/kg, mean dose of xylazine at 1.19 ± 0.26 mg/kg). T0 indicates the moment after induction before intubation. T1 represents the moment immediately following intubation, with subsequent time points recorded at 5–8 min intervals. Asterisks denote statistical differences between the KX and AX groups: *p<0.05, **p<0.01, ***p<0.001

**Fig. 4:**
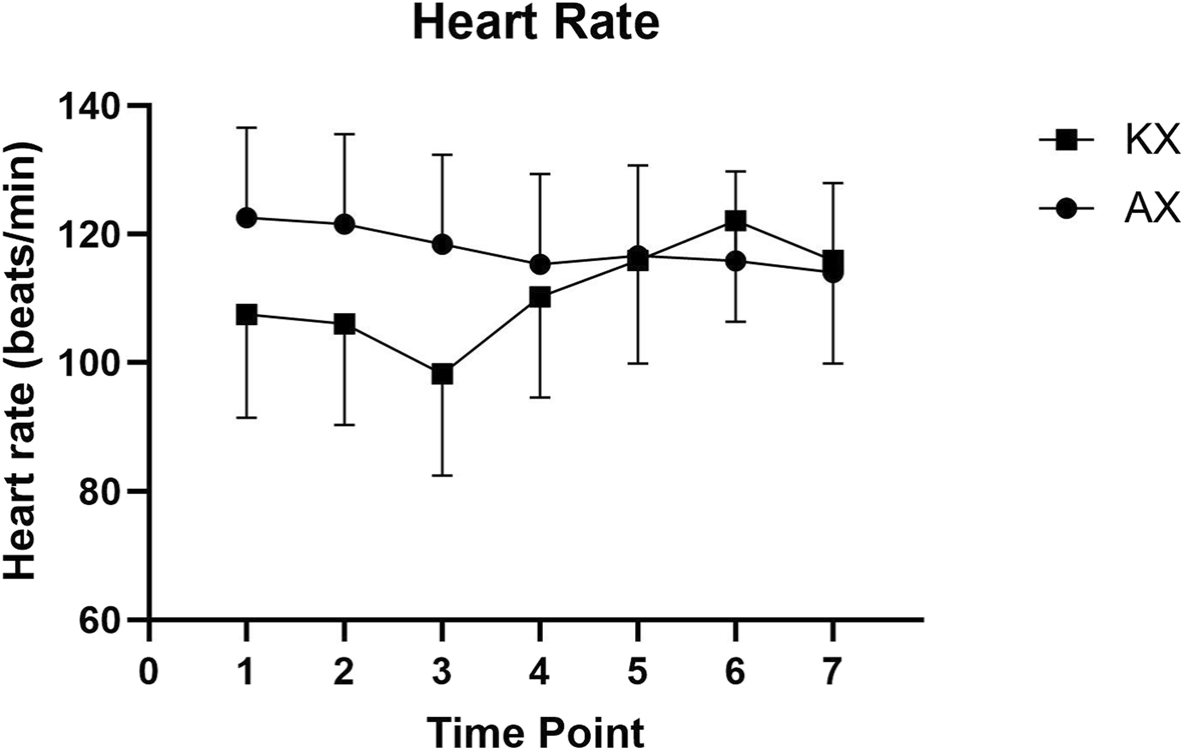
Heart rate of Reeve’s Muntjac administered intramuscular ketamine-xylazine (KX) (*n* = 4, mean dose of ketamine at 4.68 ± 2.18 mg/kg, mean dose of xylazine at 3.22 ± 1.33 mg/kg) and alfaxalone-xylazine (AX) (*n* = 5, mean dose of alfaxalone at 4.38 ± 0.31 mg/kg, mean dose of xylazine at 1.19 ± 0.26 mg/kg). T0 indicates the moment after induction before intubation. T1 represents the moment immediately following intubation, with subsequent time points recorded at 5–8 min intervals. No significant statistical differences were observed between the two groups at any time point

**Fig. 5:**
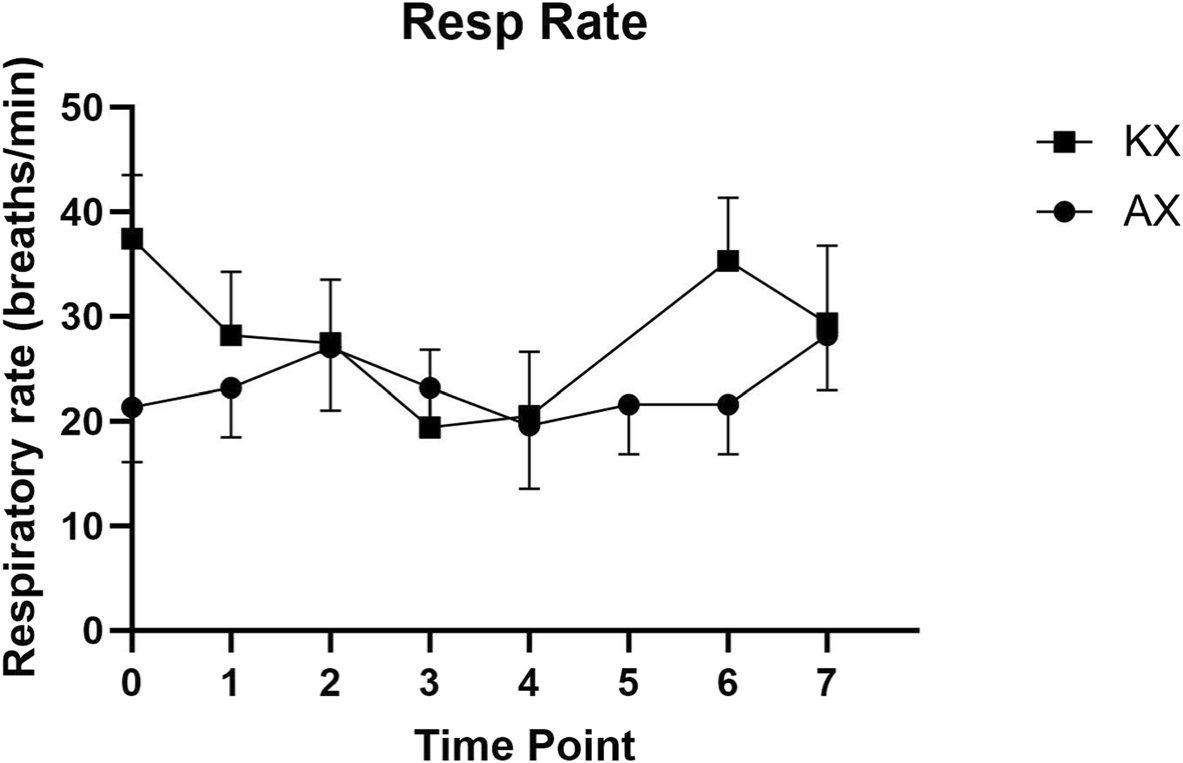
Respiratory rates of Reeve’s muntjac administered intramuscular ketamine-xylazine (KX) (*n* = 4, mean dose of ketamine at 4.68 ± 2.18 mg/kg, mean dose of xylazine at 3.22 ± 1.33 mg/kg) and alfaxalone-xylazine (AX) (*n* = 5, mean dose of alfaxalone at 4.38 ± 0.31 mg/kg, mean dose of xylazine at 1.19 ± 0.26 mg/kg). T0 indicates the moment after induction before intubation. T1 represents the moment immediately after intubation, with subsequent measurements taken at 5–8 min intervals. No statistically significant differences were observed between the two groups at any time point

**Fig. 6:**
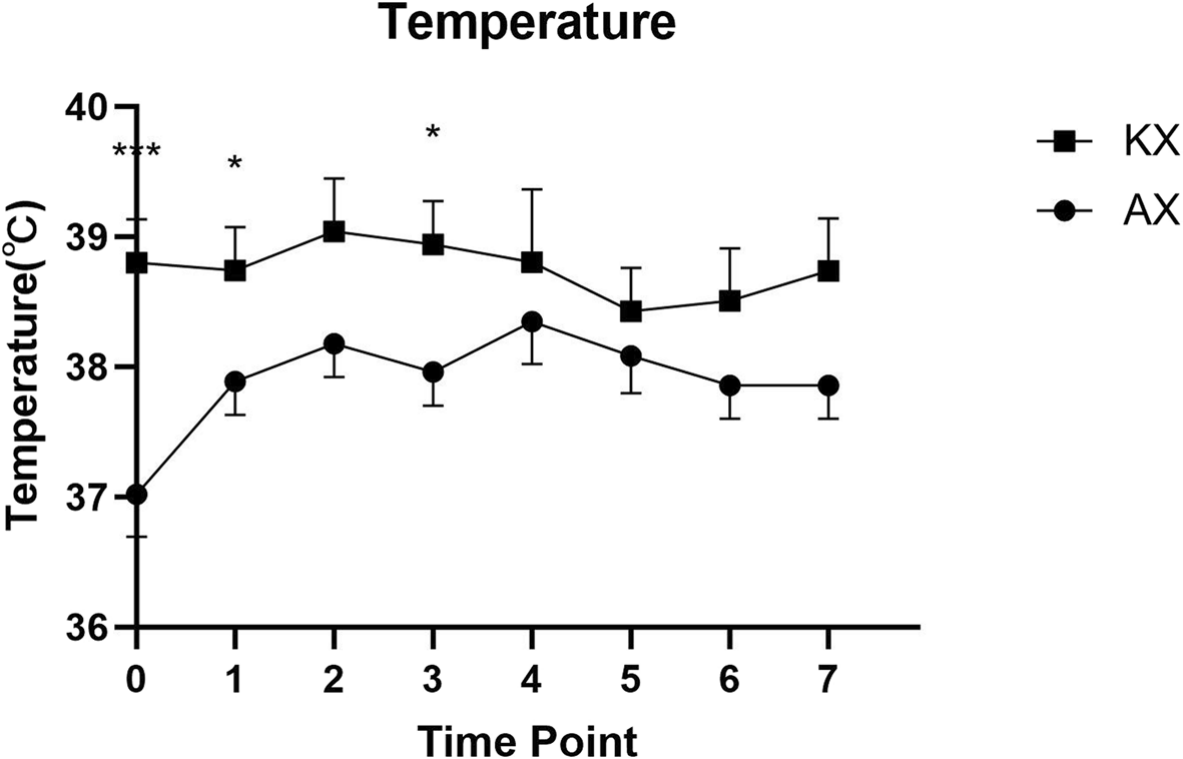
Rectal temperature measurements in Reeve’s muntjac administered intramuscular ketamine-xylazine (KX) (*n* = 4, mean dose of ketamine at 4.68 ± 2.18 mg/kg, mean dose of xylazine at 3.22 ± 1.33 mg/kg) and alfaxalone-xylazine (AX) (*n* = 5, mean dose of alfaxalone at 4.38 ± 0.31 mg/kg, mean dose of xylazine at 1.19 ± 0.26 mg/kg). T0 indicates the moment after induction before intubation. T1 represents the moment immediately following intubation, with subsequent time points recorded at 5–8 min intervals. Asterisks indicate statistical differences between the KX and AX groups: *p < 0.05, **p < 0.01, ***p < 0.001

A significant difference in SpO_2_ (*p* < 0.05) was observed between the KX and AX groups at all time points, except at T5 (*p* = 0.1074) (Fig. 3). However, there were no significant differences in heart rate or respiratory rate between the groups at any time point (Figs. 4 and 5). Rectal temperatures were significantly higher in the KX group at T0 (*p* = 0.0005), T1 (*p* = 0.0499), and T3 (*p* = 0.0253) when compared to those in the AX group (Fig. 6).

## Discussion

Deer are highly excitable animals with pronounced stress responses to external stimuli [3]. Excitement and struggle during capture and immobilization not only reduce the effectiveness of anesthetics but also cause severe complications, including fractures, capture myopathy, and death [39]. There is limited information on the anesthetic effects and selection of anesthesia protocols in Reeve’s muntjac, which impose risks and uncertainties in managing and caring for these animals in both zoos and the wild [40]. In the present study, the KX and AX combinations achieved smooth and rapid induction of anesthesia within 10 min, and the tracheas of all muntjacs were successfully intubated on the first attempt. Both protocols also provided sufficient anesthetic depth for Formosan Reeve’s muntjacs to be transported. Furthermore, noninvasive procedures, such as physical examinations, blood sample collection, and radiographs could be easily performed when either protocol was combined with isoflurane maintenance. Although there were no statistically significant differences between groups, the AX combination resulted in clinically faster induction and recovery than the KX combination.

Xylazine has been extensively used in veterinary medicine for its sedative, analgesic, and muscle relaxation effects [41–46]. Ruminants are generally more sensitive to the effects of alpha-2 agonists compared to other mammals [47, 48]. In the late 1990s, xylazine was used as the sole chemical restraint agent to immobilize Reeve’s muntjac and other deer species. However, the anesthetic effects of xylazine alone were highly unpredictable, with significant individual variability [38]. It has been reported that xylazine alone cannot induce anesthesia in Axis deer, whereas the addition of ketamine produces reliable anesthesia with acceptable cardiopulmonary function [19]. The combination of xylazine and ketamine has been shown to alleviate the undesirable adverse effects of the latter, such as hyperexcitement and muscle rigidity, while producing adequate anesthesia for wildlife immobilization [17]. The results of this study support the validity of the ketamine-xylazine combination, although the doses of xylazine and ketamine used in Reeve’s muntjac were higher than those used in other cervid species [19, 20, 41]. However, the doses used herein were similar to those in previous research on the ketamine-xylazine combination for Formosan sika deer anesthesia [10]. The muntjacs in the AX group received a lower dose of xylazine compared to the KX group. This difference was primarily due to the increased injection volume of alfaxalone, which required a reduction in the volume of xylazine to fit within a 5-ml syringe for darting. Despite the lower xylazine dose in the AX group potentially contributing to shorter recovery times as well as improved recovery scores, no statistically significant differences were observed.

Alfaxalone has been used to induce anesthesia in many species. When combined with sedative drugs, the alfaxalone combination has a shorter duration of action than ketamine combination [49], which might be another explanation why Reeve’s muntjacs in the AX group showed clinically better and faster recovery than those in the KX group. However, again, no statistically significant difference in induction and recovery parameters were detected between the groups. These results support that both alfaxalone and ketamine can provide reliable and predictable anesthesia when combined with appropriate sedatives or tranquilizers, as shown in in various species including dogs, cats, horses and pigs [50–54].

The results of the current study demonstrated that peripheral oxygen saturation was significantly higher in the AX group compared to the KX group, indicating better peripheral oxygenation with alfaxalone administration than with ketamine administration. SpO_2_ remained above 90% in the AX group throughout the procedure, while hypoxemia (SpO_2_ < 90%) was observed in the KX group at all time points except at T3 (~ 10 min post-intubation). This finding aligns with a previous study showing superior oxygenation with alfaxalone-medetomidine compared to ketamine-diazepam in sheep [55]. Alfaxalone-based combinations are known to better preserve cardiopulmonary function compared to ketamine-based combinations, maintaining well-balanced SpO_2_, mean arterial pressure (MAP), and end-tidal CO_2_ (ETCO_2_) levels [49]. In contrast, ketamine has been found to induce dose-dependent hypoventilation due to its central respiratory suppressive effect, particularly when used in combination with other sedatives [32]. The KX combination caused moderate to severe hypoxemia in white-tailed deer, which could be mitigated with oxygen supplementation via a face mask [56]. Therefore, oxygen supplementation is essential during anesthesia. In this study, oxygen was provided either by insufflation or directly via an endotracheal tube to ensure adequate oxygenation for both groups. However, hypoxemia was significantly less severe in the AX group compared to the KX group. Although the doses of xylazine were not directly compared between the groups, the dose in the KX group (3.22 ± 1.33 mg/kg) was higher than that in the AX group (1.19 ± 0.26 mg/kg). Alpha-2 agonists, including xylazine, are known to induce hypoxemia in ruminants, especially in goats and sheep [57]. Furthermore, it has been reported that ketamine combined with an alpha-2 agonist compromises the cardiac index and delivery of oxygen in sheep in sternal recumbency [58]. In addition, higher dose of xylazine in the KX group might have induced more profound vasoconstriction, leading to a decrease in the accuracy of noninvasive tissue oxygen monitoring [59]. Therefore, the xylazine-induced decrease in cardiac function and vasoconstriction might further explain why the muntjacs in the KX group had low SpO_2_ readings, which was not necessarily indicating hypoxemia, even after oxygen supplementation.

It has been reported that alfaxalone administration leads to an increase in heart rate [60], which is consistent with the findings of this study. The heart rate (reference range 59 ± 2.6 breaths per minute) [61] in the AX group was clinically higher than that in the KX group, although the difference was not statistically significant. The alfaxalone-induced decrease in vascular resistance may cause a more pronounced tachycardia due to the baroreflex [62], when compared to ketamine-induced tachycardia and increased vascular resistance from sympathetic stimulation [3]. Nonetheless, the higher dosage of xylazine in the KX group could be another reason in contributing low heart rate to the muntjacs in the KX group due to more profound vasoconstriction. Future studies should measure blood pressure to better understand the relationship between heart rate and vascular resistance.

Although there were no statistically significant differences in respiratory rate (reference range 11 ± 3.5 breaths per minute [61]) between groups at any time point, apneustic breathing was observed in the KX group. A mild increase in respiratory rate has been reported following ketamine-xylazine administration in Tuj rams [63]. The higher dose of xylazine used in the KX group might have contributed to the observed variability in respiratory patterns, making the respiratory rate less predictable. In a previous study on the hypoxemic effects of alpha-2 agonists in sheep, an increase in respiratory rate and a decrease in PaO_2_ were noted after xylazine administration [57].

The rectal temperature was significantly higher in the KX group compared to the AX group after induction. Throughout the study, the rectal temperature in the AX group was slightly lower or at the low end of the normal temperature range (37.5–39.7 ℃) for deer species [64]. This finding aligns with the sympathomimetic effect of ketamine, which can induce muscle rigidity and hyperthermia [32]. Furthermore, our results are consistent with those of a study reporting that white-tailed deer experienced an increase in rectal temperature as a primary physiological response to tiletamine-zolazepam, ketamine, and xylazine anesthesia [17]. Nevertheless, the dosage of xylazine was higher in the KX group compared to the AX group which might induce a more profound peripheral vasoconstriction to prevent body temperature redistribution and better preserve body temperature than the AX combination [65]. There was a trend of increase in rectal temperature in muntjacs receiving the AX combination, which might reflect the effect of using a warming device to prevent hypothermia. Although no detrimental hyperthermia was observed with either drug combination in this study, the risk should be closely monitored by measuring rectal temperature throughout the procedure, especially when ketamine is part of the anesthesia protocol.

Although the injection volumes were not compared between the groups in this study, the injection volume of the AX combination was subjectively larger than that of the KX combination, necessitating a 5-ml syringe for darting, while the KX combination required only a 3-ml syringe. The increased volume could reduce darting accuracy. Additionally, delivering the AX combination was more challenging due to the heavier dart. Recently, a novel 40 mg/mL concentration of alfaxalone (Jurox Pty Ltd, NSW) was released in Australia, which could reduce the injection volume of alfaxalone-based combinations, making remote delivery more feasible in larger animals and potentially reducing complications such as muscle injury [34].

The effect of xylazine was successfully reversed by 4 mg/kg IV tolazoline in all animals without significant complications during recovery. The route of administration of tolazoline was determined by the veterinarian based on the clinical signs of the individual. All animals received tolazoline intravenously after the procedure in this study because of different levels of low SpO_2_ readings (SpO_2_ < 93%); therefore, the veterinarian in charge decided to reverse xylazine as quickly as possible.

This study has several limitations. First, blood pressure measurement and blood gas analyses, including PaO_2_ and PaCO_2_ data, were not performed, which may limit the precise assessment of hemodynamics and ventilation status. Second, ETCO_2_ readings, which are crucial for evaluating ventilation, were not measured due to monitor limitations. Third, complete baseline information on physiological parameters before induction was not available, which would have improved monitoring and the comparison of post-induction changes. Despite the Reeve’s muntjacs being captive in a zoo, obtaining baseline readings without stress was challenging due to their temperament. Fourth, the dosages used in this study were not standardized, leading to different pharmacodynamic profiles between groups. The difference in xylazine dose was likely the primary factor contributing to most of the observed differences between groups. Finally, only nine Formosan Reeve’s muntjacs were included in this study, resulting in a small sample size. Several non-significant results suggest that the study may not have had sufficient power to detect meaningful differences in some outcomes, which could be a practical limitation in the study of endemic wildlife.

In summary, both the KX and AX combinations administered intramuscularly via blow dart provided rapid induction of anesthesia in Formosan Reeve’s muntjacs without significantly affecting HR, RR, and RT. However, the KX combination resulted in higher incidence of low SpO_2_ readings. Formosan Reeve’s muntjacs recovered smoothly from anesthesia with both combinations following the administration of tolazoline. To the best of our knowledge, this is the first study to use an alfaxalone-based combination to induce anesthesia in Formosan Reeve’s muntjac. Both combinations effectively induced immobilization to facilitate transportation and endotracheal intubation in these animals.

## Data Availability

Raw data supporting the conclusions of this study are available from the authors upon request.
